# Home Delivery Practices and Associated Factors in Ethiopia

**Published:** 2019

**Authors:** Ayele Gebeyehu Chernet, Kassahun Trueha Dumga, Kebadu Tadesse Cherie

**Affiliations:** 1- Statistics Department, College of Natural and Computational Sciences, Wolkite University, Wolkite, Ethiopia; 2- Department of Statistics, Wolkite University, Wolkite, Ethiopia

**Keywords:** Deliver care, Developing country, Ethiopia, Home delivery, Mixed effect, Random effect

## Abstract

**Background:**

The risk of a woman in a developing country dying from a maternal-related cause is higher compared to a woman living in a developed country. Despite the fact that delivery care service utilization is essential for further improvement of mothers and newborns, the coverage of delivery service in Ethiopia is still near to the ground. This study aimed to identify factors associated with home delivery among women in Ethiopia at their last birth.

**Methods:**

The data was obtained from 2016 Ethiopia Demographic and Health Survey which is the fourth survey. The sample was selected using a stratified, two-stage cluster sampling design and the data was analyzed using mixed effect logistic regression model.

**Results:**

A total of 10,622 women were considered in this study and 67.2% of them gave birth at home. The percentage of home delivery at their last birth was high in Afar and Somali region (89.6% and 81.7%, respectively) while only 3.3% women who lived in Addis Ababa delivered at home. Living in rural areas, being uneducated, older age, not watching TV, and being poor are predictors of home delivery at 5% level of significance.

**Conclusion:**

There is a need of giving special attention to women living in rural area, women from poor families and uneducated women to decrease home delivery.

## Introduction

Delivery care through access to health facilities and skilled health personnel is the main important intervention for safe motherhood. Historically, increasing women’s access to health facilities with the capacity to provide emergency obstetric care has been the cause for large drops in maternal mortality (1).

Despite the international emphasis in the last few years on the need to address the unmet health needs of pregnant women and children, progress in reducing maternal mortality has been slow. According to the World Health Organization (WHO), approximately 800 women die every day from preventable causes related to pregnancy and childbirth with 99% of all maternal deaths occurring in developing countries. The risk of a woman in a developing country dying from a maternal-related cause during her lifetime is about 33 times higher compared to a woman living in a developed country (2).

According to findings from levels and trends in the use of maternal health services in developing countries, except for a few countries (Benin, Namibia, Zimbabwe and Vietnam), the use of skilled care for delivery is considerably lower in sub-Saharan Africa and South/Southeast Asia than other regions. In 2011, nearly half of all women who have died due to pregnancy-related causes were from sub-Saharan Africa (3). Skilled birth attendance is one of the main interventions to combat such deaths, prompting WHO to advocate universal skilled birth attendance (4).

The age and parity are also determinants for the place of delivery. The study done in Zambia showed that 55% of women with delivery in health facilities were younger and out of that, 65% were those having their first baby. Women with 35 years and above with more than five children tend to deliver at home because they considered themselves experienced so they don’t need assistance from skilled health workers. This is evidenced by a study conducted by Mrisho in southern part of Tanzania and the study conducted in Nepal and both documented that multi Para and older women tend to deliver at home than young women. These young women have no experience in child births and they tend to fear complications related to pregnancy and child birth (5–7).

Research conducted in Nepal showed that more than half (58.1%) of the mothers had institutional delivery and 41.9% of them had home delivery. The most common reason for home delivery was easy and convenient environment (66.7%) and that for institutional delivery was safety (77.8%). There was a significant association between caste, education of mothers, education of spouse, occupation of spouse, per capital income, time to reach the nearest health center, parity, previous place of delivery, number of antenatal visit, knowledge about place of delivery with place of delivery (8).

With a maternal mortality ratio of 673 per 100,000 live births and 19,000 maternal deaths annually, Ethiopia is a major contributor to the worldwide death toll of mothers. According to 2011 Ethiopian demographic and health survey (EDHS), only 10% of births have been delivered at a health facility (9% in a public facility and 1% in a private facility). Nine women in every ten have delivered at home. Delivery at health facility was doubled from 5% to 10% when compared with 2005 EDHS (9).

Research conducted in Bahir Dar in 2014 using logistic regression model showed that 78.8% of women gave birth to their current child at health institutions. According to this research, place of residence, current age of women, employment status of both women and husband, education level of both spouses had significant contribution to mother’s delivery in the institution (10).

Delivery service by a skilled assistant is a crucial issue in reducing the risk of complications and infections that can cause death or serious illness to mothers and the newborns. Despite the fact that delivery service utilization is essential for further improvement of mothers and newborns, the coverage of delivery service in Ethiopia is still near to the ground. Even if there is physical access to institutional delivery services, many mothers may not use them because of different factors at individual, household, and community levels that shape the ability to seek health care.

Moreover, previous studies for various countries were considered about modeling only the fixed effects of covariates. Thus, the little magnitude of this service and lack of appropriateness of the model applied for clustered data have generated interest in assessing determinant factors affecting delivery care service utilization of mothers by fitting a statistical model that can explain the data in the most meaningful manner. Delivery care service utilization is quite different across regions due to different customs, culture and practice of people in Ethiopia.

Therefore, this study tried to fill the gaps by identifying factors associated with delivery at home compared to health institution in Ethiopia using mixed effect logistic regression model by considering region of respondent as a cluster.

## Methods

### 

#### Source of data and study design:

The data was obtained from 2016 EDHS, which was taken from Central Statistical Agency (CSA). It is the fourth survey conducted in Ethiopia as part of the worldwide project (11). The 2016 EDHS sample was stratified and selected in two stages. Each region was stratified into urban and rural areas, yielding 21 sampling strata. Samples of Enumeration Areas (EAs) were selected independently in each stratum in two stages. Implicit stratification and proportional allocation were achieved at each of the lower administrative levels by sorting the sampling frame within each sampling stratum before sample selection, according to administrative units in different levels, and by using a probability proportional to size selection at the first stage of sampling. After excluding missing values, a total of 10,622 women in reproductive age were included in this study from nine regional states and two city administratives.

### Study variables:

#### Response variable:

The response variable of this study was place of delivery of mothers at their last birth which was coded as 0 if a mother gave birth at health institution and 1 if at home.

#### Explanatory variables:

Based on literature, the explanatory variables included are explained in table 1.

**Table 1.** Description of explanatory variables used in the analysis

**Variable**	**Description**	**Category**
**Age**	Age of mother	15–19, 20–24, 25–29, 30–34, 35–39, 40–44, 45–49
**Education**	Education level of mother	Uneducated, primary, secondary and higher
**Wealth index**	Wealth index of the family	Poor, middle, rich
**TV**	Frequency of watching TV	Not at all, sometimes, always
**Radio**	Frequency of listening to radio	Not at all, sometimes, always
**ANC visit**	Frequency of ANC visit	Not at all, 1 up to 3 times, 4 and above times
**Residence**	Mothers’ place of residence	Rural, urban
**Abortion history**	History of terminated pregnancy	Yes, no
**Region**	Region of mothers	Tigray, Afar, Amhara, Oromia, Somali, Benishangul, SNNPR, Gambela, Harari, Addis Ababa, Dire Dawa

#### Method of data analysis:

Based on the valid data obtained, a descriptive analysis was performed using frequency and percentage for both dependent and independent variables. To identify risk factors, mixed effect logistic regression model was used. Mixed effect logistic regression model enabled us to control for dependence in data collected among respondents who lived in the same region (Clusters) and it allowed us to control the effect of unmeasured/unobserved variation at the cluster level.

Data cleaning, management and analysis were carried out using STATA, version 12. Variables were re-coded to meet the desired classification. All hypotheses to determine differences, associations and relationships were judged with the significant at p<0.05.

#### Availability of data and material:

The dataset was demanded and retrieved from DHS website after formal online registration and submission of the project title and detailed project description.

#### Ethical consideration:

Ethics approval and consent to participate Ethics approval and participant consent were not necessary as this study involved the use of a previously-published de-identified database by Central Statistical Agency of Ethiopia.

## Results

In this study, a total of 10,622 women in reproductive age were included from nine regional states and two city administrations. Based on the result given in table 2, 7,137 (67.2%) of them gave birth at home and the remaining 3485 (32.8%) delivered at health institutions. About 8648 (81.4%) lived in rural while 1974 (18.6%) lived in urban areas. Among women lived in rural areas, 77.9% of them delivered at home and in urban areas, 20.4% of them delivered at home.

**Table 2.** Basic covariates of place of delivery for last birth

**Variable**	**Category**	**Count**	**Place of delivery for last birth**		

			**Health institution**	**Home**	**p-value**
**Religion**					
	Orthodox	3077 (29.0%)	1502(48.8%)	1575 (51.2%)	<0.001
	Protestant	1858 (17.5%)	550 (29.6%)	1308 (70.4%)	
	Muslim	5436 (51.2%)	1387(25.5%)	4049 (74.5%)	
	Other	251 (2.4%)	46(18.3%)	205 (81.7%)	
**ANC visit**					
	Not at all	2481 (34.5%)	508 (20.5%)	1973 (79.5%)	<0.001
	1 to 3 times	2112 (29.4%)	404(19.1%)	1708 (80.9%)	
	4 and above	2601 (36.1%)	499 (19.2%)	2102 (80.8%)	
**Residence**					
	Urban	1974 (18.6%)	1572 (79.6%)	402 (20.4%)	<0.001
	Rural	8648 (81.4%)	1913 (22.1%)	6735 (77.9%)	
**History of abortion**					
	No	9717 (91.5%)	1913 (22.1%)	6735 (77.9%)	0.159
	Yes	905 (8.5%)	316 (34.9%)	589 (65.1%)	
**Education level**					
	Uneducated	6820 (64.2%)	1312 (19.2%)	5508 (80.8%)	<0.001
	Primary	2677 (25.2%)	1246 (46.5%)	1431 (53.5%)	
	Secondary and higher	1125 (10.6%)	927 (82.4%)	198 (17.6%)	
**Wealth Index**					
	Poor	5756 (54.2%)	957 (16.6%)	4799 (83.4%)	< 0.001
	Middle	1466 (13.8%)	424 (28.9%)	1042 (71.1%)	
	Rich	3400 (32.0%)	2104 (61.9%)	1296 (38.1%)	
**Work status**					
	No	7667 (72.2%)	2253 (29.4%)	5414 (70.6%)	0.142
	Yes	2955 (27.8%)	1232 (41.7%)	1723 (58.3%)	
	15–19	402 (3.7%)	179 (44.5%)	223 (55.5%	
**Age of mother**					
	20–24	2167 (20.4%)	822 (37.9%)	1345 (62.1%)	<0.001
	25–29	3160 (29.5%)	1045 (33.1%)	2125 (66.9%)	
	30–34	2352 (22.1%)	731 (31.1%)	1621 (68.9%)	
	35–39	1694 (16%)	516 (30.5%)	1178 (69.5%)	
	40–44	646 (6.1%)	157 (24.3%)	489 (75.7%)	
	45–49	201 (2.1%)	35 (17.4%)	166 (82.6%)	
	Not at all	8396 (79%)	1901 (22.6%)	6495 (77.4%)	
**Frequency of watching TV**					
	Sometimes	877 (85.6%)	433 (49.4%)	444 (50.6%)	<0.001
	Always	1349 (12.7%)	1151 (85.3%)	198 (14.7%)	
	Not at all	8087	2190 (27.1%)	5897 (72.9%)	
**Listening to radio**					
	Sometimes	1273	610 (47.9%)	663 (52.1%)	<0.001
	Always	1262	685 (54.3%)	477 (45.7%)	

Among women who had Antenatal Care (ANC) visit, about 2178 (46.2%) of them told about pregnancy complication and about 2534 (53.8%) of them did not tell anything to health professionals during ANC visit. About 80.8% of uneducated women gave birth at home while only 17.6% of women with a secondary education and above delivered at home. According to their wealth index, about 83.4% of poor women delivered at home while 62% of women from rich families delivered at health institutions.

The result in table 3 showed that about 3.3% of mothers from Addis Ababa city administration and 41.3% from Dire Dawa city administration delivered at home. On the other hand, 89.6% from Afar and 81.7% from Somali regional states delivered at home.

**Table 3.** Place of delivery by regional states

**Regions**	**Place of delivery for last birth**		**Total**	**p**

	**Health institution**	**Home**		
**Tigray**	596 (57.7%)	437 (42.3%)	1033	<0.001
**Afar**	110 (10.4%)	952 (89.6%)	1062	
**Amhara**	259 (26.5%)	718 (73.5%)	977	
**Oromia**	309 (19.5%)	1272 (80.5%)	1581	
**Somali**	275 (18.3%)	1224 (81.7%)	1499	
**Benishangul**	224 (25.5%)	655 (74.5%)	879	
**SNNPR**	371 (29.1%)	906 (70.9%)	1277	
**Gambela**	259 (36.9%)	442 (63.1%)	701	
**Harari**	315 (52.1%)	290 (47.9%)	605	
**Addis Ababa**	446 (96.7%)	15 (3.3%)	461	
**Dire Dawa**	321 (58.7%)	226 (41.3%	547	
**Total**			10622	

### Mixed effect logistic regression results:

Based on the result in table 4, educated women had less chance to deliver at home compared to uneducated women. The odds of delivery at home among women with primary education and secondary education and above was 0.48 and 0.28 compared with the odds of illiterate women, respectively. This means that better educated women are less likely to deliver at home compared to low educated women. Women in the middle (OR=0.61, CI: 0.4868–0.7331) and rich (OR=0.48, CI: 0.3869–0.5731) wealth qunitiles were less likely to deliver at home compared to those in the poor quintile. Rural residents were 3.47 times as likely as their urban counterparts to deliver at home (OR=3.47, CI: 2.6468, 4.2932).

**Table 4.** Results of mixed effect logistic regression model for delivery at home

**Variable**	**Category**	**OR**	**95% CI for OR**	**p-value**
**Wealth index**				
	Poor	Ref		
	Middle	0.61	[0.4868,0.7331]	<0.001
	Rich	0.48	[0.3869,0.5731]	<0.001
**Radio**				
	Not at all	Ref		
	Sometimes	1.14	[0.8674, 1.4126]	0.278
	Always	1.10	[0.8305, 1.3695]	0.455
**TV**				
	Not at all	Ref		
	Some times	0.60	[0.4471, 0.7529]	<0.001
	Always	0.31	[0.2195, 0.4005]	<0.001
**Age**				
	15–19	Ref		
	20–24	1.37	[0.8920, 1.8480]	0.073
	25–29	1.98	[1.2931, 2.6670]	< 0.001
	30–34	2.00	[1.2787, 2.7213]	<0.001
	35=39	1.95	[1.2276, 2.6724]	<0.001
	40–45	2.64	[1.4499, 3.8301]	<0.001
	45–49	4.13	[1.8192, 7.4408]	<0.001
**ANC**				
	Not at all	Ref		
	1 up to 3 times	1.02	[0.8421, 1.1979]	0.856
	4 and above times	0.96	[0.8019, 1.1181]	0.662
**Mother Education**				
	Uneducated	Ref		
	Primary	0.48	[0.4000, 0.5600]	<0.001
	Secondary & higher	0.28	[0.1971, 0.3629]	<0.001
**Residence**				
	Urban	Ref		
	Rural	3.47	[2.6468, 4.2932]	<0.001

**Random-effects**	Variance =0.64	[0.3928148 1.042745]		
**Parameters**	SE=0.159			

LR test *vs*. logistic regression, chibar2 (01)=157.33, Prob>=chibar2<0.001, Ref.=Reference, SE: Standard error

Based on the frequency of watching TV, the odds of home delivery for women watching TV were sometimes 0.60 times less than women who did not watch TV at all and for women who have watched TV were always 0.31 times less than women who did not watch TV at all. According to ages of mother, older age increases the odds of delivery at home. The odds of delivery at home for mothers having the age of 45–49 years were 4.13 times more than mothers with the age of 15–19 years. The odds of home delivery for mothers who were 45–45 years were 2.64 times more than mothers aged 15–19 years. In addition, the likelihood ratio test of mixed effect logistic regression versus logistic regression was highly significant (p=0.000) which shows there is significant heterogeneity within and between regions at the place of delivery of mothers.

## Discussion

The main goal of this study was to identify the determinants of place of delivery of women at their last birth in Ethiopia using mixed effect logistic regression model considering region of respondents as a clustering variable. Factors included in this study were wealth index, frequency of listening to radio, frequency of watching TV, age of mother, frequency of ANC visit, mothers’ level of education, place of residence, history of terminated pregnancy (Abortion history), work status of women, information about pregnancy complication, sex of household head and the outcome variable of interest was place of delivery in the last birth.

The result of the study showed that about 67.2% of mothers gave birth at home and the remaining 32.8% delivered at health institution. This result shows that there is improvement from 2011 EDHS result which included only 10% of mothers who delivered at health institutions and the remaining 90% delivered at home (9). This improvement may be due to the great emphasis given by the government to reduce maternal mortality by pregnancy complication. Research conducted in Bahir Dar in 2014 revealed that about 78.8% of mothers delivered at health institutions (10). This huge difference may come from the fact that the study had considered only the capital of Amhara regional state (Bahir Dar) where mothers are assumed to have better access to health institution and good awareness about the advantages of institutional delivery. But great difference was revealed from research conducted in Nepal which showed that more than half (58.1%) of the mothers had institutional delivery and 41.9% of them had home delivery (8).

The result of mixed effect logistic regression model revealed that living in rural areas, being uneducated, older age, not watching TV, and being poor are predictors of home delivery at 5% level of significance.

Women who lived in urban areas had better experience of institutional delivery than rural women. This result may be due to rural inhabitants usually having no or little access to maternal health and family planning programs as compared to urban residents (13). Consistent result is documented in research conducted in Amhara regional Sate (10).

According to age of mothers, older women have high probability of delivery at home compared to younger women. This result may be due to the fact that they consider themselves as having experience so they don’t need assistance from skilled workers. But young women had no experience in child births and they tend to fear complications related to pregnancy and child birth. Consistent results were also found by a research conducted in Zambia, Tanzania and Nepal. All of these findings revealed that older women tend to deliver at home compared to younger women (5–8).

The result also showed that women in the middle and rich wealth quintiles were less likely to deliver at home compared to those in the poor quintiles. This may be due to the reason that even though skilled delivery services are provided freely by government institutions in Ethiopia, there may be directly and indirectly associated costs that women in the richest families can afford. In broad terms, financial capability of the family and costs of a delivery including transportation costs may not be afforded by women among poor families. This result is consistent with a research conducted in Ethiopia which reported that proportion of births attended by skilled birth attendants in health institutions among women in the richest quintile was about 5.11 times higher than that of women in the poorest quintile (14).

Based on the level of education of mothers, there was a significant difference between educated and uneducated women on place of delivery. According to this finding, the probability of delivering at home was found to be greater for uneducated women than educated. This difference may be due to the fact that educated women are also considered to have improved knowledge and attitude and skilled maternity services are provided for them and they have the benefit in using such services. Consistent result is also documented by a research conducted in Bahir Dar and Nepal which reported that the percentage of home delivery is greater for uneducated women than the educated women (8, 9).

## Conclusion

Despite the fact that the government tried to lower the rate of home delivery by promoting the importance of institutional delivery, the rate of home delivery is still high in Ethiopia. A total of 10,622 women in the reproductive age were considered in this study from which 67.2% gave birth at home. About 77.9% of rural women and 20.4% of urban women delivered at home. About 89.6% of women living in Afar region and 81.7% of women living in Somali region gave birth at home while only 3.3% of women who lived in Addis Ababa delivered at home.

The clustering effect was significant and mixed effect logistic regression model was found to be appropriate for the given data. Based on these results, the government needs to focus on socioeconomic enhancement to be able to reduce home delivery. Most predictors are routed in lower socio-economic situation. Hence, improving education, income, access to media, and greater access to free services for institutional delivery can be effective strategies to reduce home delivery.
